# Supplementary material for: The Diagnostic Performance of Various Clinical Specimens for the Detection of COVID-19: A Meta-Analysis of RT-PCR Studies
Source: Diagnostics (Basel). 2023 Sep 26;13(19):3057. doi: 10.3390/diagnostics13193057 (PMC10572802; doi:10.3390/diagnostics13193057)
Supplement: Supplementary file 1 [file diagnostics-13-03057-s001.zip › Supplementary file S1_Search strategy.pdf]

Supplementary file S1: The detailed search strategy in various databases

A. PubMed search strategy

Search number	Query	Results
#1	Polymerase Chain Reaction, Reverse Transcriptase[Title/Abstract] OR Reverse Transcriptase PCR[Title/Abstract] OR PCR Reverse Transcriptase[Title/Abstract] OR Transcriptase PCR, Reverse [Title/Abstract] OR RT-PCR [Title/Abstract]	160383
#2	RT-PCR[Title/Abstract] OR Reverse Transcriptase Polymerase Chain Reaction [Title/Abstract]	170187
#3	#1 OR #2	175125
#4	COVID 19[Title/Abstract] OR SARS-CoV-2 Infection[Title/Abstract] OR 2019 Novel Coronavirus Disease[Title/Abstract] OR 2019-nCoV Disease[Title/Abstract] OR Coronavirus Disease-19[Title/Abstract]	294446
#5	COVID-19	333562
#6	#4 OR #5	333562
#7	#3 AND #6	8384

B. Scopus search strategy

Search number	Query	Results
#1	TITLE-ABS-KEY Polymerase Chain Reaction, Reverse Transcriptase) OR TITLE-ABS-KEY Reverse Transcriptase PCR) OR TITLE-ABS-KEY PCR Reverse Transcriptase) OR TITLE-ABS-KEY Transcriptase PCR, Reverse) OR TITLE-ABS-KEY RT-PCR	261367
#2	TITLE-ABS-KEY RT-PCR) OR TITLE-ABS-KEY Reverse Transcriptase Polymerase Chain Reaction	59745
#3	#1 OR #2	256173
#4	ITLE-ABS-KEY COVID 19) OR TITLE-ABS-KEY SARS-CoV-2 Infection) OR TITLE-ABS-KEY 2019 Novel Coronavirus Disease) OR TITLE-ABS-KEY 2019-nCoV Disease) OR TITLE-ABS-KEY Coronavirus Disease-19)	388498
#5	#4 AND #5	12653

C. Cochrane search strategy

#1	MeSH descriptor: [COVID-19] explode all trees	MeSH	2590
#2	RT-PCR OR Reverse Transcriptase Polymerase Chain Reaction	Limits	4049
#3	#1 AND #2	Limits	154

D. Embase search strategy

History Save | Delete | Print view | Export | Email Combine using And Or Collapse

#4	#3 AND 'article'/it	10,815
#3	#1 AND #2	15,132
#2	'reverse transcription polymerase chain reaction'	346,720
#1	'coronavirus disease 2019'/exp OR 'coronavirus disease 2019'	299,653
