# Supplementary material for: The Diagnostic Performance of Various Clinical Specimens for the Detection of COVID-19: A Meta-Analysis of RT-PCR Studies
Source: Diagnostics (Basel). 2023 Sep 26;13(19):3057. doi: 10.3390/diagnostics13193057 (PMC10572802; doi:10.3390/diagnostics13193057)
Supplement: Supplementary file 1 [file diagnostics-13-03057-s001.zip › Supplementary file S2_Rv1_Characteristics of RTPCR.pdf]

Supplementary file S2: Characteristics of the RT-PCR techniques

Study ID	Sample used	Sample storing	Viral RNA isolation	RT-PCR procedure
Escobar DF et al.,	Saliva and NPS	at -20°C	Using the ExiPrep 96 Viral DNA/RNA Kit and the automated system ExiPrep™ 96 Lite (BIONEER, Daejeon, South Korea)	Using the 7,500 Fast Real-Time PCR System thermocycler (Applied Biosystems, CA, USA)
Singh J et al.,	NPS and OPS	at -80 ◦C	Using HiPurAViral RNA purifcation kit manufactured by HiMedia Laboratories Pvt. Ltd	Using TaqPath COVID-19 Combo Kit, LabGun COVID-19RT-PCR Kit 94, Allplex 2019-nCoV Assay ; BGI Kit Real-Time Fluorescent RT-PCR Kit for Detecting SARSCoV-2 And TRUPCR Kit
Figueroa S et al.,	NPS	NR	processed with AccuPrep Viral RNA extraction kit IVD (Bioneer, South Korea)	High Capacity Superscript cDNA Reverse Transcription Kit (Applied Biosystems, USA) for retrotranscription (RT) and DreamTaq Green PCR Master Mix (Termo Scientifc, USA) for PCR
LeGof J et al.,	Saliva and NPS	at 4 °C	MGIEasy® Nucleic Acid Extraction Kit (MGI Tech Co, Shenzhen, China)	e TaqPath™ COVID-19 CE IVD RT PCR Kit
Villota SD et al.,	NPS and sputum:	NR	Qiagen Viral RNA Mini Kit), and the Trizol LS Reagent (Invitrogen)	Through (DA0930; Da An Gene Co. Ltd. of Sun Yat-sen University) and SuperScript™ III Platinum™ One-Step RT-qPCR Kit (Invitrogen) kit
De Pace V et al.,	LRT specimens (bronchoalveolar lavage and Broncho aspirates)	at – 80 °C	by means of an NX-48 viral nucleic acid extraction kit (Genolution, Seoul, South Korea).	RT-PCR testing by means of the Allplex™ SARS-CoV-2 assay (Seegene Inc., South Korea) and followed by e Vivalytic SARS-CoV-2 rapid PCR assay (Bosch Healthcare Solutions GmbH, Germany)
Kanwar N et al.,	Residual NPS	at -70˚C	by NucliSENS easyMAG (bioMerieux, Inc; Marcy-l'Étoile, France); m2000sp extractor by Abott;	Abbott Real Time SARS-CoV-2 assay (Abbott assay), Aptima™ SARS-CoV-2 assay (Aptima assay), BGI Real-Time SARS-CoV-2 assay (BGI assay), Lyra® SARS-CoV-2 assay (Lyra assay), and DiaSorin Simplexa™
Michel J et al.,	Dry or wet NPS and/or OPS	At −40 to −80 °C	using the QIAamp Viral RNA Mini Kit	Two duplex one-step real-time RT-PCR assay (orf1ab assay)

Wu S et al., 2021; China	Throat, nasal, NPS and sputum samples	NR	Through High Pure Viral Nucleic Acid kit (Roche Diagnostics, Mannheim, Germany).	Five RT-qPCR kits (ORF1ab/N) named B to F through ABI 7500 Real Time PCR system (Applied Biosystems, Foster City, CA)
Lee KK et al.,	Combined nasal and throat swab	NR	NucliSENS® easyMag® (bioMérieux) platform	in-house RT-PCR<br>the AllplexTM2019- nCoV Assay from SeeGene (Seoul, South Korea; 6%)
Borkakoty et al.,	NPS or OPS	-20 °C	using nucleic acid a commercial spin-column based extraction kit (QIAamp Viral RNA Mini Kit, Qiagen, Hilden, Germany)	A Type Specific Primer based one step RT-PCR (TSP-PCR)
Hata DJ et al.,	NPS and saliva	-30 °C	Roche MagNa Pure LC 2.0 (Roche Diagnostics, Indianapolis, IN)	Cobas 6800 System, hereafter cobas (Roche Molecular Systems, Branchburg, NJ)
Wang B et al.,	Throat swab and faecal sample	−80 °C	NP968-S Nucleic Acid Extraction System (Tianlong Science & Technology Co., Ltd, Xi'an, China) with paramagnetic particle method, using the Virus DNA/RNA Isolation Kit (Tianlong Science & Technology Co., Ltd, Xi'an, China)	Seven kits including kit A (BGI Biotech Co., Ltd, Wuhan, China), kit B (Outdo Biotech Co., Ltd, Shanghai, China), kit C (Sansure Biotech Inc., Changsha, China), kit D (Perkin Elmer Medical Diagnostic Products, Co., Ltd, Shanghai, China), kit E (Daan Gene Co., Ltd. Of Sun Yat-Sen University, Guangzhou, China), kit F (Jiangsu Bioperfectus Technologies Co., Ltd, Taizhou, China), and kit G (Fosun Long March Medical Science Co., Ltd, Shanghai, China).
Mollaei HR et al.,	NPS	NR	MWG Thermo cycler System (MWG, Germany)	Nucleocapsids (N), Envelope (E), RNA depended RNA Polymerase (RdRp), ORF1ab and Spike (S) primers through in-house conventional RT-PCR using One-Step supermix RT-PCR Master Mix (Biosystem, Germany)
Pierri B et al.,	NPS	−80 °C	Automated platform (GeneQuality X120, AB Analitica, Italy) with a magnetic bead-based protocol, using the GeneQuality X120 Pathogen kit (AB Analitica)	Real-time RT-PCR by RQ-2019-nCoV kit (AB Analitica) and Bio-Rad's QX200 system (Bio-Rad Laboratory, Hercules, California, USA) for digital droplet RT-PCR

Torres A et al.,	Sputum	NR	NR	RT-qPCR test (CDC 2019-Novel Coronavirus Real-Time RT-qPCR Diagnostic Panel upper and lower respiratory specimens)
Pearson JD et al.,	NPS	−80 °C	Qiagen RNeasy, Invitrogen Purelink, Norgen Biotek Total RNA Purifcation Kit and the BGI Magnetic Bead Viral RNA/DNA extraction kit	The 2019-nCoV TaqMan RT-PCR Kit from Norgen Biotek and 2019-nCoV: Real-Time Fluorescent RT-PCR kit from BGI
Kriegova E et al.,	Painless nasal-swab self-collection	4 °C	Automated nucleic acid magnetic bead extraction platform, Zybio EXM 3000 (Zybio, Shenzhen, China), and a nucleic acid extraction kit (Zybio).	Novel Coronavirus (2019-nCoV) Real-Time Multiplex RT-PCR Kit (LifeRiver, Shanghai, China)
Onyilagha C et al.,	Residual NPS	-70 °C	MagMAX™ CORE Nucleic Acid Purifcation Kit (CORE kit)	Biomeme RRT- PCR assay (CDC EUA assay)
Desmet T et al.,	OPS/NPS	NR	BioMérieux, Marcy-l'Étoile, France	Qiagen One Step RT-PCR Kit, Cat. number 210212, Qiagen, Hilden, Germany
Kanji JN et al.,	NP and Deep nasal turbinate swab	−70 °C	Microlab STARlet (Hamilton Company, Reno, USA); NucliSENS EasyMAG (BioMérieux, Marcy-L'Etoile, France); MagMAX (ThermoFisher Scientific, Waltham, USA)	Centers for Disease Control (Atlanta, USA) SARS-CoV-2 assay (Integrated DNA Technologies, Coralville, USA); RNAse P rtRT-PCR kit (Integrated DNA Technologies, Coralville, USA)
Gómez-Romero L et al.,	Saliva	4°C	QIAamp viral RNA mini kit (QIAGEN)	StarQ One-Step RT-qPCR (Genes 2 Life) kit
Milosevic J et al.,	NPS	−80°C	Direct-zolTM RNA Microprep (R2060, Zymo Research)	ultrafast one-step qRT-PCR assay using CFX96 Real-Time PCR detection system (Bio-Rad Laboratories)
Pekosz A et al., 2021; United States	NPS	−70°C	Qiagen viral RNA isolation kit	Quidel Corporation. Athens, Ohio
Ferreira BLS et al., 2021; Brazil	NPS/serum/saliva	-80°C	QIAamp Viral RNA Mini Kit (Qiagen, Hilden, Germany)	High Resolution Melting analysis (HRM-RTqPCR) using Applied Biosystems Quantstudio 3 Real-Time PCR System (ThermoFisher, Waltham (Massachusetts), USA and HOT FIREPol EvaGreen HRM Mix (No ROX)

Dumaresq J et al.,	Natural spring water gargle, ONPS	-18ºC	No RNA extraction	Direct RT-PCR using Allplex™ 2019-nCoV Assay (Seegene)
Morecchiato F et al.,	NPS	4°C	Extraction free protocol	SARS-CoV-2 (Allplex™ SARS-CoV-2 assay and Allplex™ SARS-CoV-2/FluA/FluB/RSV assay)
Olearo F et al.,	Gargle	NR	NR	cobas SARS-CoV-2 IVD test for the cobas6800 system (Roche, Mannheim, Germany)
Ghoshal U et al.,	NPS	NR	QIAamp RNA mini kit (Qiagen, Inc., Valencia, Calif.)	TrueNat Chip based RT-PCR by Studio 5 Real Time PCR system (Thermo Fisher Scientific, Massachusetts USA)
Balaska S et al.,	Saliva	2–8 °C	NeumoDxTM SARS-CoV-2 Assay	Advanta Dx SARS-CoV-2 RT-PCR Assay (Fluidigm Corporation, South San Francisco, CA, USA)
Watanabe Y et al.,	NPS	NR	Smart Gene (Mizuho Medy Co., Ltd.)	real-time qRT-PCR- based point-of-care test using N2 primers and detection by a quenching probe (QProbe, Mizuho Medy Co., Ltd.)
Domnich, A et al., 2021; Italy	NPS	NR	STARMag Universal Cartridge Kit (Seegene Inc., South Korea) on an automated Nimbus IVD (Seegene Inc., South Korea) platform	CFX96™ instrument (Bio-Rad Laboratories, USA) using an Allplex™ 2019-nCoV assay (Seegene Inc., South Korea)
Kim YK et al., 2021; South Korea	NPS	NR	Real-Prep Viral DNA/RNA kit (BioSewoom Inc., Seoul, Korea)	Allplex 2019-nCoV assay (Seegene, Seoul, Korea)
Micocci, M et al., 2021; UK	NPS	NR	Integrating magnetic bead-based nucleic acid extraction	POCKIT™ Central Nucleic Acid Analyzer (POCKIT™ Central)
Carvalho RF et al., 2021; Brazil	OPS	2-8°C	QIAamp Viral RNA Mini Kit, Qiagen, Valencia, CA, USA	2019-NCOV_N Positive Control Kit, Integrated DNA Technologies, Coralville, IA, USA; 200,000 copies/μL
Kritikos A et al., 2021; Switzerland	NPS or Saliva	NR	MagNA Pure 96 instrument (Roche)	Automated Cobas 6800® system (Roche-Switzerland)
Brotons P et al., 2021; Spain	Saliva	-80°C	GeneFinder COVID-19 Plus RealAmp kit, Elitech, France	GeneFinder COVID-19 Plus RealAmp kit, Elitech, France;
Laverack M et al., 2021; USA	NPS and AN		MagMAX Viral/Pathogen II Nucleic Acid Isolation Kit (Applied Biosystems,	EZ-SARS-CoV-2 Real Time RT-PCR assay (Tetracore, Inc., Rockville, MD) and the TaqPath

			Foster City, CA) and a KingFisher Flex Magnetic Particle Processor	COVID-19 Combo Kit Multiplex Real-Time RT-PCR assay (Thermo Fisher Scientific Inc.)
Avetyan et al.,	NPS and RNA samples	NR	Maxwell RSC Viral Total Nucleic Acid Purification Kit (Promega Corporation Inc, US) [5 samples]; triazole based PREP-NA kit DNA Technologies Ltd, Russian Federation [114 samples] and magnetic bead-based ZipPrime nucleic acid isolation kit (ZipPrime Ltd, Turkey) [111 samples]	Novel Coronavirus (2019-nCoV Nucleic Acid Diagnostic Kit, Sansure Biotech, China and SARS-CoV-2/SARS-CoV, DNA technology, Russia
Hernandez MM et al.,	Saliva	-80°C	chemagicTM Viral DNA/RNA 300 Kit H96 (CMG-1033-S; PerkinElmer) on the automated chemagicTM 360 instrument (2024-0020; PerkinElmer)	MassARRAY® SARS-CoV-2 Panel and MassARRAY® System (CPM384; Agena)
Hernández C et al.,	NPS	NR	NR	9 RT-PCR kits including QuantuMDx SARS CoV 2 RT PCR Detection Assay by QuantuMDx; GeneFinder™ COVID-19 Plus RealAmp Kit by GeneFinder; Allplex™ 2019-nCoV Assay by Seegene; MiRXES Fortitude Kit 2.1 by MiRXES; Coronavirus COVID-19 genesig® Real-Time PCR assay by Genesig; Novel Coronavirus (2019-nCoV) Nucleic Acid Diagnostic Kit by Sansure; Smart Detect™ SARS-CoV-2 rRT-PCR Kit by Inbios; ProTect™ COVID 19 PCR Kit JN by Medsys and PCL COVID19 Speedy RT-PCR by PCL
Tastanova A et al.,	NPS and/or OPS	-80C	magnetic beadebased (SpeedBeads, GE Healthcare, Darmstadt, Germany) extraction kit for the KingFisher instrument (MagMax, Thermo Fisher Scientific, Waltham, MA)	a) CDC 2019-Novel Coronavirus Real-Time RT-PCR Diagnostic Panel (for in vitro diagnostics); b) Applied Biosystems TaqMan 2019-nCoV Assay Kit version 1; c) Applied Biosystems Multiplex TaqMan 2019- nCoV Assay Kit version 2; d) EURORealTime SARS-CoV-2; e) Real-time RT-PCR assays for the detection of SARS- CoV-2, Pasteur Institute, Paris, France, and f) In-house customized RT-PCR protocol

Leber W et al.,	Venous blood	NR	NR	SARS-CoV-2 RT-qPCR using Roche LightCycler using a primer set provided by TIB MOLBIOL
Gadkar VJ et al.,	NPFS	NR	QIAsymphony (Qiagen, Hilden, Germany) automated extraction platform, using the DSP Virus/Pathogen kit (Qiagen)	4X TaqMan Fast Virus 1-step Master Mix (ThermoFisher: cat No 4444434) on the ABI Fast 7500 real-time PCR system (ThermoFisher, CA)
Bruno et al.,	NPS	NR	PureLink Viral RNA/DNA Mini Kit (Invitrogen, USA) using CFX96 BioRad instrument	SARS-CoV-2 RT-PCR using CDC protocol (de "Charite protocol)
Sun R et al.,	NPS	NR	NR	RT-PCR
Rigo S et al.,	NPS	NR	STARMag 96 X 4 Universal Cartridge Kit (Seegene Inc.)	Laboratory practice (RNA-Extraction followed by RT-PCR), or, in parallel, by-passing RNA-extraction step (Direct RT-PCR) on Seegene's automated platform (Allplex SARS-CoV-2 Assay).
Banko A et al.,	NPS	-80 ◦C	Viral DNA/RNA Extraction Kit (ALPHAGENE Co. Ltd., Sengnam, Korea). on the automatic nucleic acid extraction system NC-15 plus (ALPHAGENE Co. Ltd.),	Three kits including GeneFinderTM COVID-19 Plus RealAmp Kit (OSANG Healthcare Co., Seongnam, Korea), Sansure Biotech(Sansure Biotech Inc., Changsha, China), and TaqPathTM COVID-19 CE-IVD RT-qPCR Kit (Thermo Fisher Scientific, Waltham, MA, USA)
Noor AK et al.,	NPS	-20 °C	QIAamp Viral RNA Mini Kit (Qiagen, Germantown, USA)	Singleplex and Multiplex RT-PCR method uses the N1, N2 and P gene
Fitoussi F et al.,	NPFS	NR	EX3600 extractor (Liferiver and Shanghai ZJ Bio-Tech Co., Ltd)	The VitaPCR™ SARS-CoV-2 Assay performed on the VitaPCR™ Instrument (Credo Diagnostics Biomedical Pte. Ltd.; distributed in France by Biosynex)
Freire-Paspuel B et al.,	NPS	-80°C	AccuPrep Viral RNA extraction kit" (Bioneer, South Korea)	AccuPower SARS-CoV-2 Multiplex RT-PCR kit (Bioneer, South Korea) and Allplex 2019-nCoV Assay (Seegene, South Korea),
Dierks S et al.,	NPS	NR	NR	Genesig Real-Time PCR Coronavirus (COVID-19) assay (Primerdesign Ltd., Chandlers Ford, UK)

Nakura Y et al.,	Sputum, NPS, Saliva	-80˚C	QIAamp Viral RNA Mini Kit (QIAGEN, Valencia, CA, US)	e QuantStudio 5 Real-time PCR system (Thermo Fisher Scientific)
Stockdale AJ et al.,	NPS	NR	Automated QIAsymphony platform (Qiagen, Germany)	commercial CE-marked in vitro diagnostic assay (Viasure SARS-CoV-2, Biotec, Spain), target sequences in the SARS-CoV-2 open reading frame (ORF)-1ab and N genes
Kortela E et al.,	NPS	NR	NR	RT-PCR testing was conducted by one of the following methods (gene targets): Cobas® SARS-CoV-2 test kit on the CobasC® 6800 system (orf1ab and E) (Roche Diagnostics, Basel, Switzerland), Amplidiag® COVID-19 test (orf1ab and N) (Mobidiag, Espoo, Finland,) and a laboratory-developed test based on a protocol recommended by WHO (N) (non-exponential amplification curves and amplification with cycle treshold values >34 in the laboratory-developed test were reanalysed with either Cobas or Amplidiag)
Altamimi AM et al.,	NPS and OPS	-80°C	xiPrep 96 Viral RNA Kit, and ExiPrep 96 Lite Automated NA Purification System (Bioneer)	Nine kits including<br>1. LightMix Modular (Roche Diagnostics, Germany; E gene & RdRP gene)<br>2. RealStar SARS-CoV-2 (E & S)<br>3. TaqPath (N, Orf1b &S)<br>4. DiaPlexQ (N & Ofr1a)<br>5. Lyra SARS-CoV-2 assay (Orf1ab (PP1ab)<br>6. BGI (Orf1ab)<br>7. KAIRA (E & RdRP)<br>8. PowerChek™ (E & RdRP)<br>9. Sansure (N &Orf1ab)<br>10. Genesig (RdRP)<br>11. IQ REAL (Orf1ab)<br>12. RADI (S & RdRP)
Visseaux B et al.,	NPS	-20 °C	No extraction	Three extraction-free RT-PCR assays such as (i) PrimeDirect® Probe RT-qPCR Mix (Takara), (ii) PrimeScript®RT-PCR (Takara), and (iii) SARS-CoV-

				2 SANSURE®BIOTECH Novel Coronavirus (Sansure)
Cassinari K et al.,	NPS and Saliva	-20°C	EZ1 DSP virus kit (Qiagen, Hilden, Germany) and EZ1 Advanced XL machine	**RT-qPCR:** RealStar® SARS-CoV-2 RT-PCR Kit 1.0 (Altona Diagnostics, Hamburg Germany), performed on a CFX96™ Real-Time PCR Detection System (BioRad, California, USA)<br>**RT-ddPCR**: One-Step RT-ddPCR Advanced Kit for Probes (Bio-Rad Laboratories, Hercules, CA, USA) on QX200 ddPCR platform (Biorad)
Carrillo RJD et al.,	NPS and snort-spit Saliva	NR	GenAmplify™ Viral RNA Purification Kit (The Manila HealthTek, Inc.)	Novel Coronavirus (2019-nCoV) Nucleic Acid Diagnostic Kit (Sansure Biotech Inc.) or Real-Time Fluorescent RT-PCR Kit for Detecting SARS-CoV-2 (BGI Genomics Co. Ltd.)
Girish P et al.,	NPS and Saliva	NR	RNA extraction kits by Qiagen, Zybio, Gsure	Real-Time RT-PCR Master Mix multiple primer and probe sets designed commercially for SARS CoV-2 (Meril Diagnostics, Lab genomics Labgun, ICMR NIV protocols)
Freire-Paspuel B et al., (2)	NPS	NR	Using AccuPrep Viral RNA extraction kit (Bioneer, Daejeon, South Korea) as an alternate RNA extraction method, and CFX96 BioRad instrument	using COVID-19 Nucleic Acid Test Kit such as from eDiagnosis and Sansure Biotech
Dong L et al.,	Pharyngeal swab	4 °C	MagMAX-96 viral RNA isolation kit (Thermo Fisher Scientific)	QX200 digital PCR platform and three different commercial RT-qPCR kits (H&R from Shanghai Huirui Biotechnology Co., Ltd, BioGerm from Shanghai BioGerm Medical Biotechnology and Daan from Daan Gene Co., Ltd)
Gupta-Wright A et al.,	NPS	NR	NR	Panther Fusion (Hologic; ORF1ab Region 1/2 target) or Abbott RealTime (RNA-dependent RNA polymerase, nucleocapsid target)
Dimke H et al.,	NPS or OPS	NR	Acid guanidinium thiocyanate-phenol-chloroform method or the automated Maxwell® RSC 48 instrument (Promega).	RT-qPCR assay

Alaifan T et al.,	NPS	-80°C	No extraction or preheating	RT-PCR kit targeting E gene
Onwuamah CK et al.,	NPS	-20°C	Qiagen Viral RNA Kit (Qiagen Inc, Valencia, CA, USA).	Five One-step RT-PCR assaysnamely the BGI, Da An Gene, Primerdesign Genesig, Liferiver and Tib MolBiol
Price TK et al.,	Mixed (NPS, Bronchoalveolar lavage, Expectorated sputum, and miscellaneous sample types)	−80°C	1. EZ1 Advanced XL (Qiagen, Hilden, Germany) or NUCLISENS easyMAG (bioMérieux, Hazelwood, MO)<br>2. LIASON MDX instrument (DiaSorin Molecular)<br>3. MagMax Viral/Pathogen Nucleic Acid Isolation Kit using the automated KingFisher Flex Purification System (Thermo Fisher Scientific)	Three RT-PCR techniques:<br>1. CDC COVID-19 RT-PCR assay<br>2. Simplexa COVID-19 Direct Real-Time RT-PCR assay<br>3. TaqPath COVID-19 RT-PCR assay (Applied Biosystems 7500 Fast Real-Time PCR instrument)
Trobajo-Sanmartín C et al.,	Saliva	- 80 °C	STARMag 96 × 4 universal extraction system (Seegene, Seoul, Korea) with the Hamilton Microlab STARlet automation robot (Hamilton Company, Reno, NV, USA)	AllplexTM 2019-nCoV assay (Seegene, Seoul, Korea) on the CFX96 real-time PCR detection system (Bio-Rad, Hercules, CA, USA) and Cobas® 6800 platform (Roche Diagnostics GmbH, Mannheim, Germany)
Omar S et al.,	Mixed	NR	NR	SARS CoV-2 Smartchecker PCR kit (Genesystem, South Korea). There were two PCR gene targets: the N (nucleocapsid) gene and the RdRp (RNA-dependent RNA polymerase) gene
Bergevin MA et al.,	NPS and saliva	NR	No extraction	Allplex 2019-nCoV Assay (Seegene)
Yip CCY et al.,	Mixed	NR	NucliSENS easyMAG extraction system (bioMérieux, Marcy-l'Étoile, France)	i) Hecin Scientific SARS-CoV-2 nucleic acid test kit, a dual-target real-time RT-PCR assay; and ii) SARS-CoV-2 N gene was performed using QuantiNova Probe RT-PCR Kit (QIAGEN, Hilden, Germany) on LightCycler 480 II Real-Time PCR System (Roche)
Renzoni A et al.,	NPS	-80° C	NucliSens easyMAG (bioMérieux, Marcy-l'Étoile, France)	SuperScript™ III Platinum™ One-Step qRT-PCR Kit (Invitrogen, Carlsbad, CA, USA) in a CFX96 Thermal Cycler (Bio-Rad, Hercules, CA, USA)

Tsujimoto Y et al.,	NPS, NS and Saliva	Below -70 °C	NR	Cobas 6800 Systems RT-PCR device (Roche Molecular Systems, South Branchburg, NJ)
Mio C et al.,	NPS	NR	ELITe InGenius® SP200 (ELITechGroup) system of SARS-CoV-2 RNA	One-Step Reverse Transcription-Droplet Digital Polymerase Chain Reaction (RT-ddPCR)
Lau YL et al.,	NPS	NR	QIAamp Viral RNA Minikit (Qiagen, Germany)	Bio-Rad CFX qPCR (Bio-Rad, USA)
Shen L et al.,	NPS	NR	Thermo Scientific™ KingFisher™ Flex Magnetic Particle Processors (cat no. KFR-805496; Thermo Fisher: Waltham, MA).	Four kits targets were ORF1ab gene, N gene, and ribonucleoprotein (RNP) from Beijing Applied Biological Technologies Co., Ltd; Beijing Kinghawk Pharmaceutical Co., Ltd; Beijing NaGene Diagnosis Reagent Co., Ltd; and Coyote Bioscience Co., Ltd
Freire-Paspuel B et al., (3)	NPS	NR	AccuPre Viral RNA extraction kit IVD" (Bioneer, South Corea)	AccuPower SARS-CoV-2 Real Time RT-PCR kit" (Bioneer, South Korea)
Guo J et al.,	OPS and sputum	-20 ± 5°C	As per instruction manual	Multiple Real-Time PCR Kit developed by Beijing Applied Biological Technologies Co.
Lu Y et al.,	Throat swab	NR	Using magnetic beads following the manufacturer's recommended protocol (Zhongyuan, Chongqing, China)	RT-PCR kit from Sansure Biotech Inc (Hunan, China; Lot No. 2 020 007) and Shanghai BioGerm Medical Biotechnology Co., Ltd. (Lot No. 20200304A) which target ORF1ab/N gene
Ramírez AM et al.,	Serum	-20◦C	automatic eMAG® Nucleic Acid Extraction System (Biomerieux, France)	Two rRT-PCR assays: cobas® SARS-CoV-2 test (cobas® test), a qualitative assay for detection of SARS-CoV-2 RNA; and TaqPath™ COVID-19 CE-IVD RT-PCR Kit (TaqPath™ test), a multiplex RT-PCR assay for qualitative detection of SARS-CoV-2 nucleic acids
Yang M et al.,	Mixed	NR	The Stream SP96 automatic nucleic acid extraction instrument (Da An Gene Co., Ltd. of Sun Yat-sen University)	Five commercial RT-qPCR diagnostic test kits i) Da An (Da An Gene Co., Ltd. of Sun Yat-sen University, Guangzhou, China; batch number 2020030); ii) Liferiver (Shanghai ZJ Bio-Tech Co., Ltd., Shanghai, China; batch number P20200512); iii) Kinghawk (Beijing Kinghawk Pharmaceutical Co., Ltd., Beijing, China; batch

				number 20200608113), iv) eDiagnosis (Wuhan Easy Diagnosis Biomedicine Co., Ltd., Wuhan, China; batch number 200606), and v) Maccura (Maccura Biotechnology Co., Ltd., Chengdu, China; batch number 0520251)
Sahoo A et al.,	OPS	-80°C	Trueprep® AUTO/AUTO v2 Universal Cartridge based Sample Prep Device and Trueprep® AUTO/AUTO v2 Universal Cartridge based Sample Prep Kit	Chip Based Real Time RTPCR (TrueNat) by TrueNatTM Beta n-CoV test
Hofman P et al.,	NPS	4°C	Idylla™ SARS-CoV-2 IUO and the Idylla TM SARS-CoV-2 CE-IVD	Idylla™ SARS-CoV-2 Test on a platform capable of fully automated nucleic acid testing including extraction, amplification, and detection in a single-use cartridge
Jamal AJ et al.,	NPS and Saliva	-80°C	NR	RT-PCR by Allplex 2019-nCoV Assay (100T) (Seegene Inc, Seoul, Korea)
Ngaba GP et al.,	Mixed	NR	No extraction	AMPLIQUICK® SARS-CoV-2 technique
Procop GW et al.,	NPS and NS	NR	MagNA Pure system (Roche)	Five RT-PCR Diagnostic Panels including CDC n-COV; TIB MOLBIOL/Roche z 480 Assay; Xpert Xpress SARS-CoV-2 (Cepheid); Simplexa COVID-19 Direct Kit (DiaSorin); and ID Now COVID-19 (Abbott)

LRT: Lower respiratory tract; NA: Not applicable; NPFS: Nasopharyngeal flocked swabs; NPS: Nasopharyngeal swab; NR: Not reported; NS: Nasal sample; OPS: Oropharyngeal swab; URT: Upper respiratory tract
