# Supplementary figures and images for: The Diagnostic Performance of Various Clinical Specimens for the Detection of COVID-19: A Meta-Analysis of RT-PCR Studies
Source: Diagnostics (Basel). 2023 Sep 26;13(19):3057. doi: 10.3390/diagnostics13193057 (PMC10572802; doi:10.3390/diagnostics13193057)

Supplementary file S2: The funnel plot for publication bias

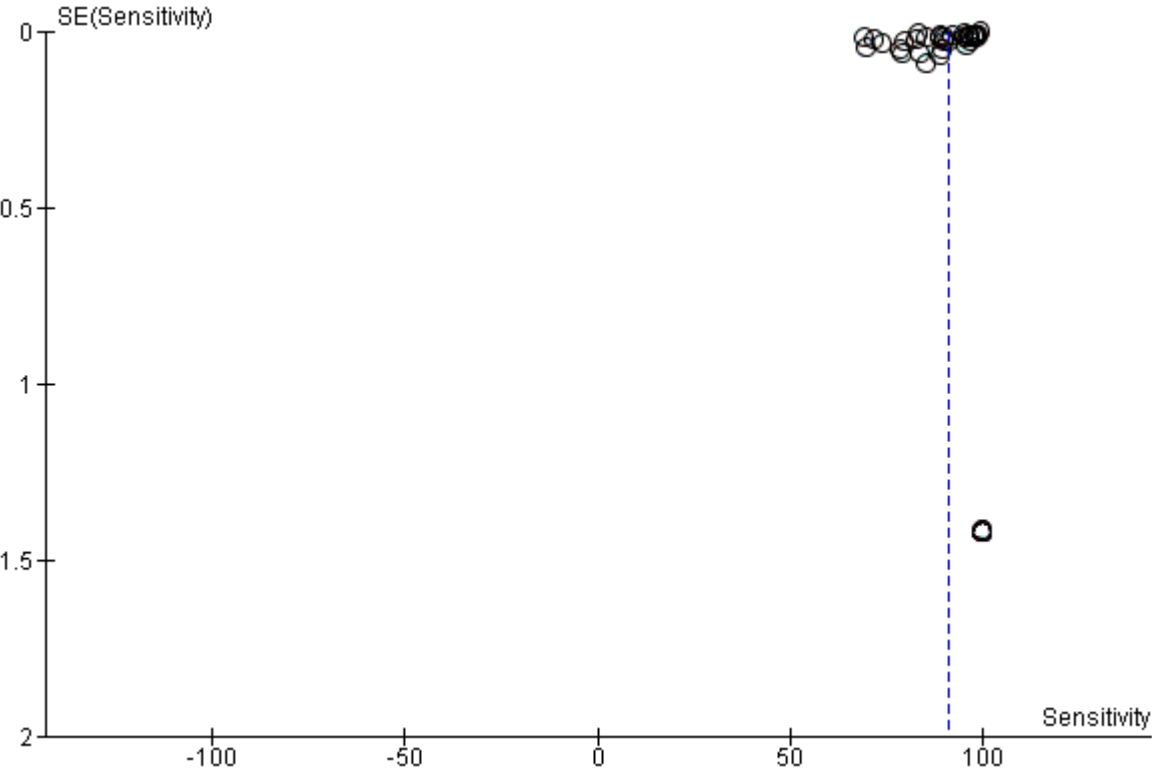

Supplement: Supplementary file 1 [file diagnostics-13-03057-s001.zip › Supplementary file S3_Funnel plot.pdf]

## Supplementary file S3: Sensitivity analysis

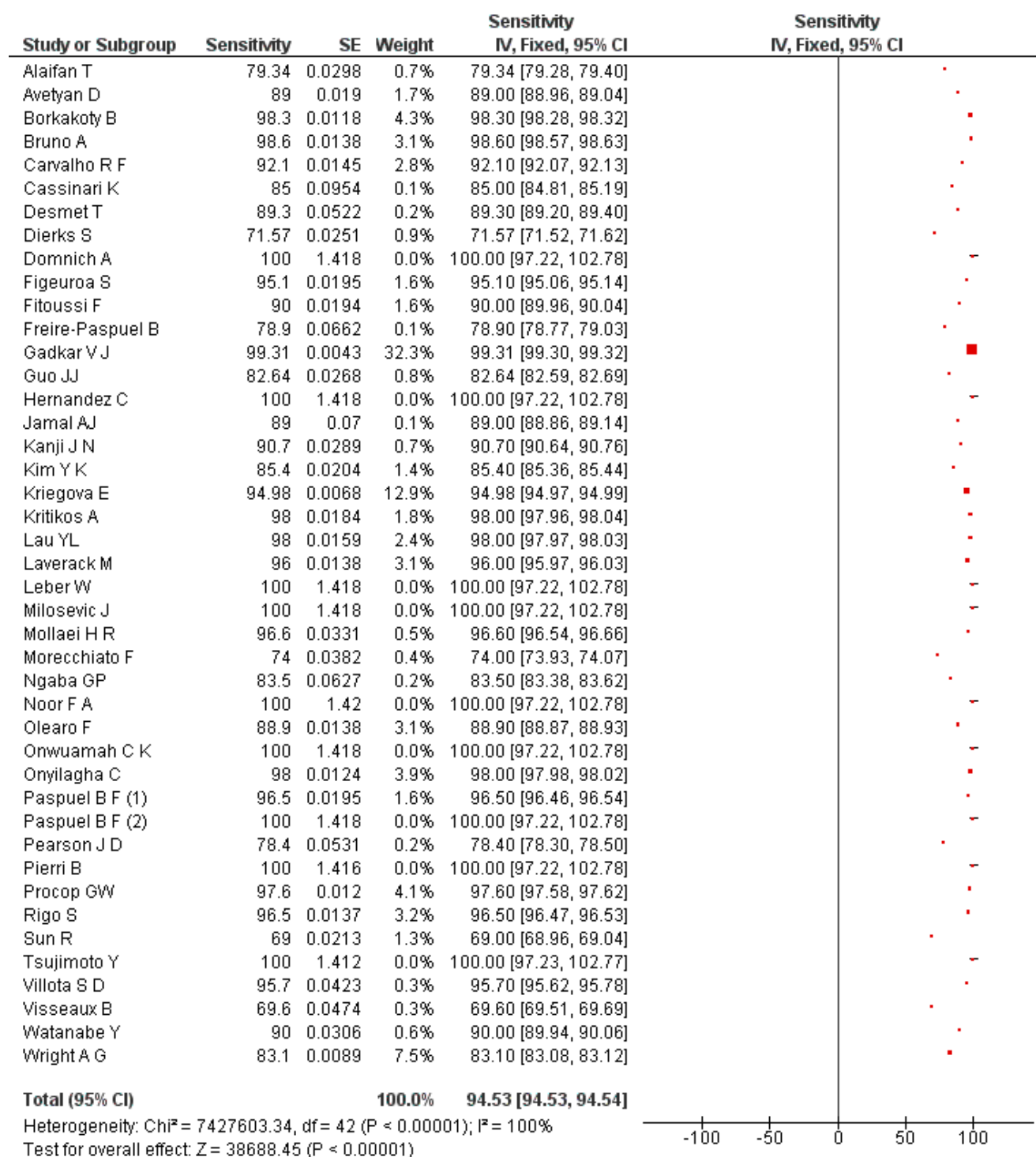

Supplement: Supplementary file 1 [file diagnostics-13-03057-s001.zip › Supplementary file S4_Sensitivity analsysi.pdf]
